# The development of evaluation scale of the patient satisfaction with telemedicine: a systematic review

**DOI:** 10.1186/s12911-024-02436-z

**Published:** 2024-02-01

**Authors:** Yifei Du, Yu Gu

**Affiliations:** Department of Medical Information Technology and Management, Yanjing Medical College, Capital Medical University, Beijing, China

**Keywords:** Telemedicine, Patient satisfaction, Scale, Systematic review

## Abstract

**Background:**

Since the outbreak of the COVID-19 pandemic, telemedicine become more and more popular, patients attempt to use telemedicine to meet personal medical needs. Patient satisfaction is a key indicator of insight into the patient experience.

**Purpose:**

This systematic review aims to explore the measurement factors of patient satisfaction with telemedicine and develop a more comprehensive and systematic scale of patient satisfaction with telemedicine.

**Methods:**

In February 2023, a literature search was conducted on the PubMed, EMBASE, and Web of Science, identifying measurement factors and tools of patient satisfaction with telemedicine. For inclusion, the studies had to have or make a questionnaire about patient satisfaction with telemedicine delivered through video/audio visits in English. The quality of the studies was evaluated according to the Critical Appraisal Tool for Analytical Cross-Sectional Studies of the Joanna Briggs Institute (JBI). The dimensions and items in each tool were also analyzed.

**Results:**

The initial search showed 14,020 studies. After eliminating duplicates and utilizing inclusion and exclusion criteria, 44 studies were included. This systematic review identified and integrated the measurement factors and develops a scale of patient satisfaction with telemedicine, which was divided into 9 dimensions and consists of 37 items.

**Conclusion:**

Future measurement and evaluation of telemedicine will benefit from scale that was developed in this study, and it will more directly reflecting patient needs when patient satisfaction with telemedicine is evaluated.

**Supplementary Information:**

The online version contains supplementary material available at 10.1186/s12911-024-02436-z.

## Introduction

The World Health Organization (WHO) defines telemedicine as “an interaction between a healthcare provider and a patient when the two are separated by distance”, and this communication may be synchronous (as in telephone or video consultations) or asynchronous (when data, queries and responses are exchanged by email or short message service) [1–3]. Telemedicine could not only provide clinical support and improve health outcomes, but also avoid patient travels, decrease exposure for patients and medical staff, and reduce health sector costs [2–5]. Therefore, It became an essential component of the medical response [6].

During the COVID-19 pandemic, telemedicine played an important role in provision of healthcare services to patients [7]. The use of telemedicine delivered through synchronous visits in various countries has increased significantly. At the early stage of pandemic, the weekly telemedicine visits have increased from 12,000 to 1,000,000 in just 3 months in the United States [8]. During the isolation period, the remote consultation involving basic medical care has reached 1.2 million people per day in the UK [8]. Telemedicine expanded tremendously and continue to flourish [9]. In other words, telemedicine has completely changed the medical service mode [10].

Patient satisfaction is one of the most significant indicators reflecting assurance of validation and acceptance of this emerging medical service mode [11]. As the voice of the patient, it is the only source of information that can report how they were treated and if the treatment patients received met their expectations [12]. With the increasing uptake of telemedicine, it is necessary to insight into what practices and process patients consider to be satisfied with [13]. However, Barsom et al. mentioned that different studies used a diverse range of questionnaires to measure patient satisfaction with telemedicine, which resulted in heterogeneous data, so it is difficult to compare and combine results of different studies [14]. Agbali et al. concluded that it was necessary to develop a standardized uniform patient satisfaction with telemedicine evaluation tool to increase versatility and agility [15]. Therefore, In this systematic review, we aimed to summarized and integrated the relevant measurement factors of patient satisfaction with telemedicine and develop a more comprehensive and systematic patient satisfaction scale for future research use.

## Methods

This systematic review was conducted in accordance with the Preferred Reporting Items for Systematic Reviews and Meta-analyses (PRISMA) statement [16]. The protocol was registered with PROSPERO under registration number CRD42022369348.

### Study design and search strategy

The retrieval formula consisted of two main parts: “telemedicine” and “satisfaction”. Through the two main parts, the following search terms were used: “telemedicine”, “telehealth”, “telecommunication”, “teleconferenc*”, “videoconferenc*”, “video consultation”, and, “satisfaction”, “experience”, “perception”, “preference”. Based on the above search terms, we tailored search strategies to each database and used controlled medical subject headings (MeSHs) and search filters where available, or Boolean search methods and free-text terms (Supplementary 1). Due to the outbreak of COVID-19 pandemic, the telemedicine audiovisual mode developed rapidly, and people paid more attention to its satisfaction. During this period, a lot of relevant research appeared, therefore, the search scope for the study was determined from January 2020 to February 2023.

### Data sources

A systematic literature search was conducted in the following databases: PubMed, EMBASE, and Web of Science. We also carried out hand searches from reference lists of retrieved studies.

### Study selection

The inclusion and exclusion criteria are described in Table 1. Two reviewers screened search results by title and abstract to identify studies whether meet the inclusion criteria outlined above. The full text of potentially eligible studies was retrieved and assessed by the two same reviewers. Any disagreement between them over the eligibility of studies was resolved through discussion with a third reviewer.

**Table 1:** Inclusion and exclusion criteria

Item	Inclusion Criteria	Exclusion Criteria
Participants	Patients who received telemedicine services.	Other populations.
Interest of phenomenon	Telemedicine delivered through video/audio visits.	Other telemedicine modes.
Outcomes	Patient satisfaction.	–
Study type	Qualitative, quantitative, or mixed methods studies.	Reviews.
Language	Only used English.	–

### Data extraction

The included studies were read in full. Two reviewers performed the relevant information and data that were collated in Microsoft Excel, which includes author, year of publication, country, study design, disease type, telemedicine mode, questionnaire dimension, and the number of satisfaction measurement factors.

### Quality and risk of bias assessment

To ensure the validity and credibility of this study, the quality of the included studies was evaluated according to the Critical Appraisal Tool for Analytical Cross-Sectional Studies of the Joanna Briggs Institute (JBI) [9]. Quality assessment of the included studies was conducted by two reviewers. Any disagreement between them over the quality assessment of literatures was resolved through discussion with a third reviewer.

## Results

### Study selection

**Fig. 1:**
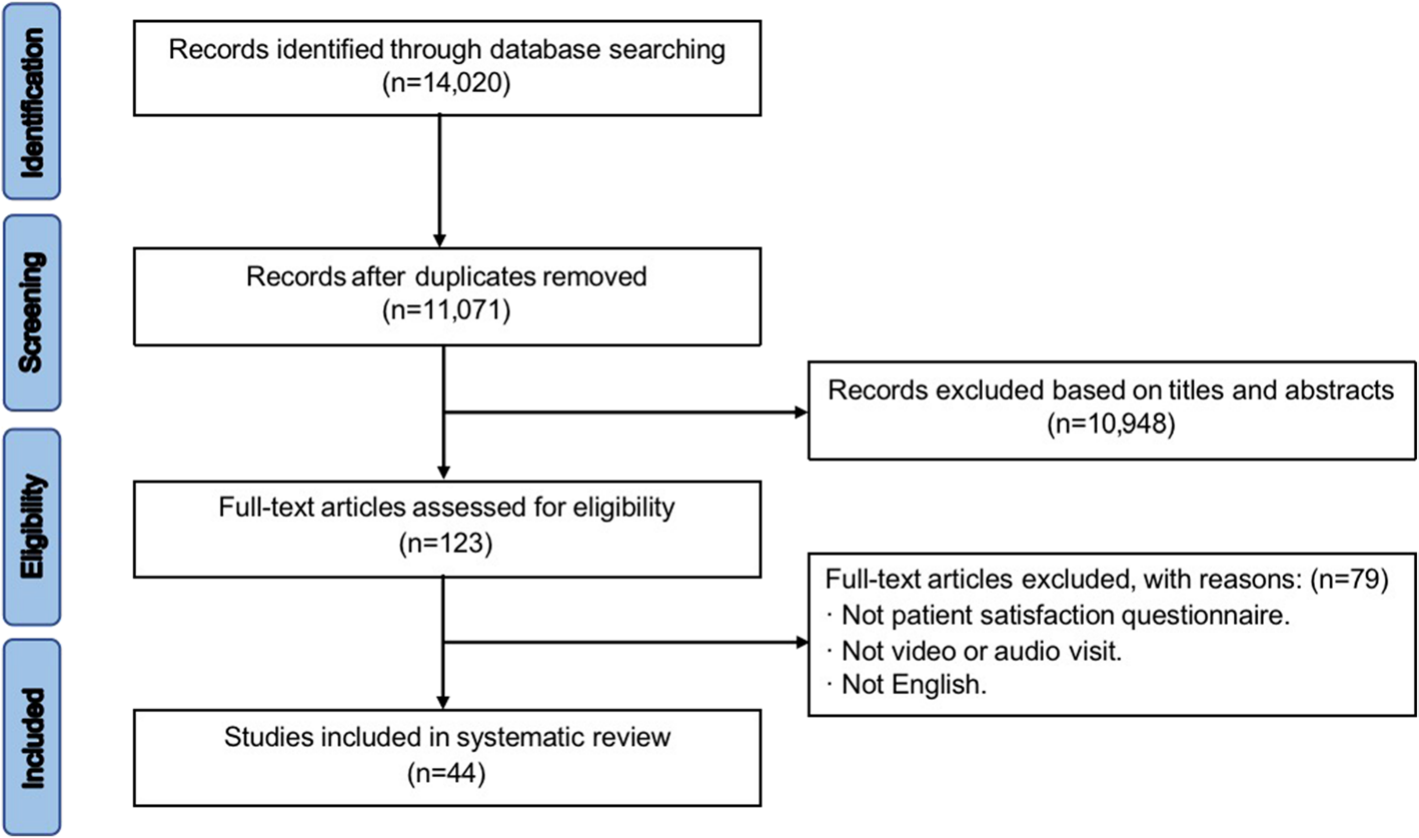
Flowchart of the selection process

The study selection process and the results of the literature search are depicted in Fig. 1. Using our search strategy, 14,020 studies were retrieved from 3 databases. After removing 2949 duplicates, 11,071 studies were screened by titles and abstracts, and 10,948 studies were excluded. The remaining 123 studies were read full text, 79 were excluded and finally 44 studies were selected for this study.

### Study characteristics

The characteristics of 44 studies were summarized in Table 2. The included studies were all the cross-sectional study, and each study was about telemedicine that delivered through video/audio visits. Most of included studies (*n* = 32) were conducted in United States [17–48], and a few were conducted in Italy (*n* = 3) [49–51], Spain (*n* = 2) [52, 53], Egypt (*n* = 1) [54], Australia (*n* = 1) [55], India (*n* = 1) [56], United Kingdom (*n* = 1) [57], Canada(*n* = 1) [58], France (*n* = 1) [59], Colombia (*n* = 1) [60]. The vast majority included studies (*n* = 38) reported on the types of participants’ diseases, which includes head and neck otolaryngology (*n* = 1) [17], pediatric (*n* = 1) [52], physical, occupational, and speech therapy (*n* = 1) [18], orthopaedic (*n* = 1) [19], pediatric pulmonary (*n* = 1) [20], cancer (*n* = 4) [21, 30, 50, 58], neurology (*n* = 1) [22], pediatric urology (*n* = 1) [24], rhinology (n = 1) [25], neuromuscular disorder (*n* = 1) [26], allergy (*n* = 1) [27], pediatric diabetes (*n* = 1) [28], epilepsy (n = 1) [56], prechemotherapy (*n* = 1) [29], pediatric rheumatology (*n* = 1) [32], neurosurgery (n = 1) [33], cystic fibrosis (*n* = 1) [35], pediatric and young adult type 1 diabetes (*n* = 1) [49], shoulder arthroscopy (*n* = 1) [36], pediatric surgery (*n* = 1) [53], vascular surgery (*n* = 1) [57], maternal mental health and substance use disorder treatment (*n* = 1) [38], referral (*n* = 1) [39], dermatology (*n* = 1) [40], endovascular neurosurgery (*n* = 1) [41], gynecologic cancer (*n* = 1) [42], orthopedic (*n* = 1) [43], urogynecology (*n* = 1) [44], ophthalmology (*n* = 1) [45], irritable bowel syndrome (n = 1) [46], sickle cell disease (*n* = 1) [47], bariatric (n = 1) [59], craniosynostosis-operated children (*n* = 1) [60], chronic neurologic disorders (n = 1) [51], and colorectal surgery (*n* = 1) [48]. While the remaining studies (*n* = 6) did not limit the patient types [23, 31, 34, 37, 54, 55]. For the evaluation questionnaire, there are part of studies (*n* = 16) using existing questionnaires [17, 22, 26, 28, 30, 32–34, 42, 46, 47, 50, 55, 56, 58, 59], a few of studies’ questionnaire (*n* = 3) were designed based on different studies [21, 24, 49], several studies (*n* = 7) evaluating by self-developed questionnaires [19, 25, 29, 39, 51, 57, 60], and some (*n* = 18) studies did not mension the specific questionnaires used [18, 20, 23, 27, 31, 35–38, 40, 41, 43–45, 48, 52–54]. In addition, the questionnaires used in several studies (*n* = 18) were divided into various dimensions [17, 20, 23, 24, 26, 32, 34, 37, 38, 44, 47, 49–51, 53–55, 57].

**Table 2:** Compilation of observations for our sample

Author, Year	Country	Disease Type	Telemedicine Mode	Evaluation Questionnaire	Questionnaire Dimension	Number of Items
Layfield et al. 2020 [17]	United States	Head and neck otolaryngology	Video visit	Telehealth Usability Questionnaire (TUQ)	5: usefulness, ease of use, effectiveness, reliability, and satisfaction	21
López Seguí et al. 2020 [18]	Spain	Pediatric	Video visit	No mention	No dimension	10
Tenforde et al. 2020 [19]	United States	Physical, Occupational, and Speech Therapy	Video/Audio visit	No mention	No dimension	7
Abdel Nasser et al. 2021 [20]	Egypt	No limited	Video visit	No mention	2: participants’ satisfaction, and attitude toward telehealth and telemedicine	13
Bate et al. 2021 [21]	Australia	No limited	Video visit	An existing questionnaire was developed by Bate et al	4: confidence, overall quality of consultation, cost saved, and time saved	4
Bisson et al. 2021 [22]	United States	Orthopaedic	Video/Audio visit	A self-developed questionnaire	No dimension	9
Capusan et al. 2021 [23]	United States	Pediatric Pulmonary	Video/Audio visit	No mention	4: technology, the experience of the visit, overall satisfaction, and likelihood to use the telehealth platform again	30
Chang et al. 2021 [24]	United States	Cancer	Video/Audio visit	Design based on prior studies	No dimension	7
Dratch et al. 2021 [25]	United States	Neurology	Video/Audio visit	Modified Telehealth Usability Questionnaire (MTUQ)	No dimension	6
Drerup et al. 2021 [26]	United States	No limited	Video/Audio visit	No mention	3: friendliness of registration staff, convenience of appointment times, and communication with physicians	9
Gan et al. 2021 [27]	United States	Pediatric Urology	Video visit	Design based on prior studies	3: a visit’s impact on access to care, patient/family experience and a visit’s effectiveness	6
Hentati et al. 2021 [28]	United States	Rhinology	Video/Audio visit	A self-developed questionnaire	No dimension	7
Hooshmand et al. 2021 [29]	United States	Neuromuscular Disorder	Video visit	The Utah Telehealth Patient Satisfaction survey	7: communication, timeliness of physician, picture quality, sound quality, protection of privacy, the comfort of the physical exam, and ease of receiving telehealth	8
Lanier et al. 2021 [30]	United States	Allergy	Video visit	No mention	No dimension	6
March et al. 2021 [31]	United States	Pediatric Diabetes	Video visit	A 12-item Parent Satisfaction Survey	No dimension	12
Nair et al. 2021 [32]	India	Epilepsy	Video visit	A 14-point Teleme dicine Satisfaction Questionnaire	No dimension	14
Sathiyaraj et al. 2021 [33]	United States	Prechemotherapy	Video visit	A questionnaire developed by study investigators	No dimension	8
Shaverdian et al. 2021 [34]	United States	Cancer	Video/Audio visit	An existing questionnaire was developed by MSKCC	No dimension	20
Volcy et al. 2021 [35]	United States	No limited	Video visit	Not mentioned	No dimension	3
Waqar-Cowles et al. 2021 [36]	United States	Pediatric Rheumatology	Video visit	Telehealth Usability Questionnaire (TUQ)	4: usefulness, ease of use, effectiveness, and satisfaction	14
Yoon et al. 2021 [37]	United States	Neurosurgery	Video visit	An existing questionnaire was developed by Hicks et al	No dimension	8
Zimmerman et al. 2021 [38]	United States	No limited	Video/Audio visit	Clinically Useful Patient Satisfaction Scale (CUPPS)	3: clinician attitude and behavior, office environment and staff, global satisfaction and expectation of improvement	14
Ahmed et al. 2022 [39]	United States	Cystic Fibrosis	Video/Audio visit	Not mentioned	No dimension	6
Bassi et al. 2022 [40]	Italy	Pediatric and Young Adult Type 1 Diabetes	Video visit	Design based on prior studies	4: adequacy of medical care, psychological impact of telemedicine, possible advantages and future use of telemedicine, and telenursing	15
Cascella et al. 2022 [41]	Italy	Cancer	Video visit	Telehealth Usability Questionnaire (TUQ)	6: usefulness, ease of use & learnability, interface quality, interaction quality, reliability, and satisfaction and future use	22
Cha et al. 2022 [42]	United States	Shoulder Arthroscopy	Video/Audio visit	Not mentioned	No dimension	8
Chen et al. 2022 [43]	United States	No limited	Video/Audio visit	Not mentioned	3: access, care provider, and overall assessment	9
Cockrell et al. 2022 [44]	Spain	Pediatric Surgery	Video visit	Not mentioned	9: provider rating, office recommendation, explaining, listening, questions, understanding, medical history knowledge, respect, and time	9
Contractor et al. 2022 [45]	United Kingdom	Vascular Surgery	Video visit	A questionnaire developed by by a team of experts	3: acceptability of teleconsultation, benefits of teleconsultation, and future role and acceptability of virtual clinic	17
Gondal et al. 2022 [46]	Canada	Cancer	Video visit	A modified existing questionnaire	No dimension	8
Guille et al. 2022 [47]	United States	Maternal Mental Health and Substance Use Disorder Treatment	Video visit	Not mentioned	4: overall quality of care, similarity to face-to-face care, access to care, and MH care	24
Jones et al. 2022 [48]	United States	Referral	Video visit	A questionnaire was developed by a QI committee based on prior studies	No dimension	2
Kaunitz et al. 2022 [49]	United States	Dermatology	Video visit	Not mentioned	No dimension	4
Majmundar et al. 2022 [50]	United States	Endovascular Neurosurgery	Video visit	Not mentioned	No dimension	7
Mojdehbakhsh et al. 2022 [51]	United States	Gynecologic Cancer	Video/Audio visit	Telehealth Satisfaction Scale (TeSS)	No dimension	11
Omari et al. 2022 [52]	United States	Orthopedic	Video/Audio visit	Not mentioned	No dimension	7
Sansone et al. 2022 [53]	United States	Urogynecology	Video/Audio visit	Not mentioned	5: scheduling, technology, provider, personal needs, and overall satisfaction	19
Summers et al. 2022 [54]	United States	Ophthalmology	Video/Audio visit	Not mentioned	No dimension	4
Yu et al. 2022 [55]	United States	Irritable Bowel Syndrome	Video/Audio visit	Telehealth Usability Questionnaire (TUQ)	No dimension	13
Zhang et al. 2022 [56]	United States	Sickle Cell Disease	Video/Audio visit	Telemedicine Satisfaction Questionnaire (TSQ)	4: interpersonal communication, caring, care delivery, and proficiency	24
Daouadji-Ghazou et al. 2023 [57]	France	Bariatric	Video/Audio visit	An existing questionnaire	No dimension	9
Kilipiris et al. 2023 [58]	Colombia	Craniosynostosis-Operated Children	Video visit	A questionnaire was developed by the surgical members of the craniofacial team	No dimension	9
Rosellini et al. 2023 [59]	Italy	Chronic Neurologic Disorders	Video visit	A questionnaire was developed by Google Moduli	3: satisfaction for current televisit, opinions about future televisit, and quality of doctor–patient relationship	11
Yao et al. 2023 [60]	United States	Colorectal Surgery	Video visit	Not mentioned	No dimension	7

### The outcome of quality assessment

**Fig. 2:**
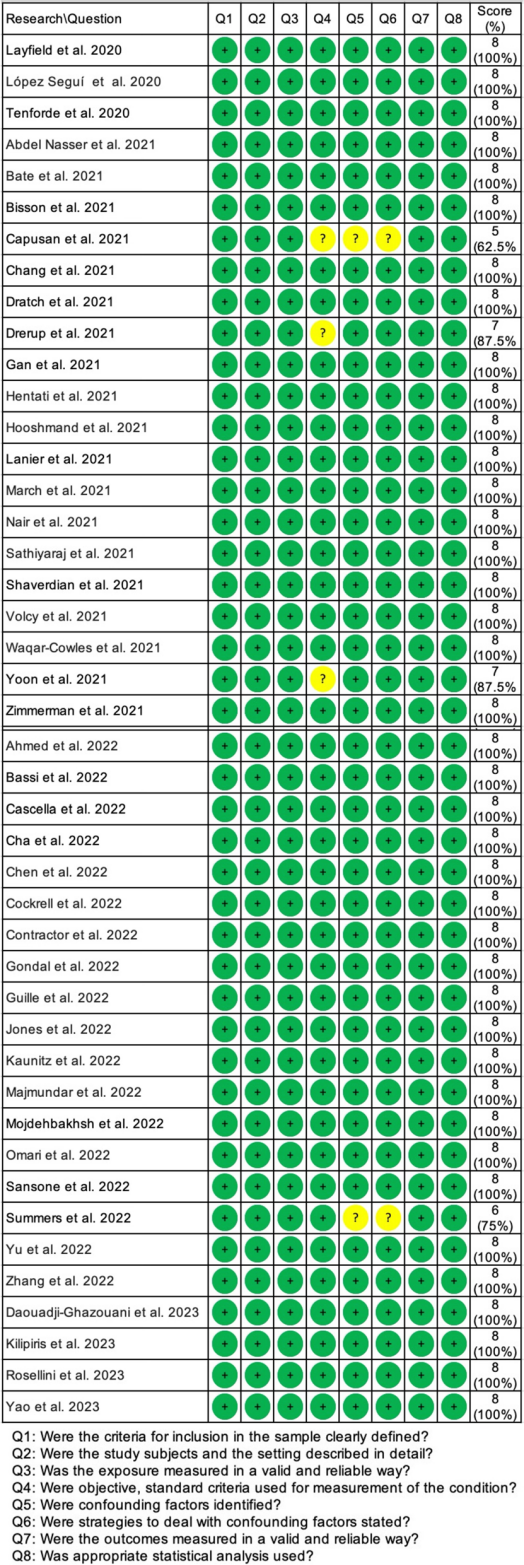
Quality assessment

Based on the Critical Appraisal Tool for Analytical Cross-Sectional Studies of the Joanna Briggs Institute (JBI), 42 studies were at low risk of bias [17–19, 21–44, 46–60], while the rest 2 studies were rated as the moderate risk of bias [20, 45]. The details of study quality are shown in Fig. 2.

### Synthesis of results

We summarized the measurement factors of patient satisfaction with telemedicine of 44 included studies. The process of integrating factors led us to find that the measurement of patient satisfaction with telemedicine involves various dimensions. To ensure the scientific rationality of this study, it is essential to build a conceptual framework for measuring patient satisfaction. 18 included studies’ questionnaires presented in Table 2 are dimensioned [17, 20, 23, 24, 26, 32, 34, 37, 38, 44, 47, 49–51, 53–55, 57]. The dimensions involve perceived usefulness and perceived ease of use of Technology Acceptance Model (TAM) [61], facilitating conditions of the model of Unified Theory of Acceptance and Use of Technology (UTAUT) [62], and interpersonal manner, technical quality, accessibility/convenience, finances, efficacy/outcomes, continuity, physical environment, and availability of a widely used Patient Satisfaction Questionnaire [63]. Based on the above category of models and literatures, we have divided all items into 9 dimensions: humanistic care, doctor-patient communication, service efficiency, diagnosis and treatment result, ease of use, system quality, usefulness, privacy and security, overall satisfaction.

According to above dimensions and items in included studies, we sorted out and combined them. Of the 44 included studies, 14 studies involved humanistic care, which includes the courtesy, friendliness, and care of doctors to patients [21, 23, 30, 33, 34, 37, 38, 42–44, 47, 49, 53, 56]. Twenty-eight studies involved doctor-patient communication, which includes doctor’s listening to patients, doctor’s explanations to patients, and communication between doctors and patients [17, 19–21, 23, 26–28, 30, 32, 34, 36–38, 40, 42, 44, 46–48, 50–54, 56, 58, 60]. Nine studies involved service efficiency, which includes the punctuality of the telemedicine visiting process [17, 26, 36, 38, 44, 48, 50, 51, 60]. Twenty-three studies involved diagnosis and treatment result, which includes whether patients’ problems, concerns, and needs were achieved [17, 18, 20–22, 24, 25, 32, 34, 38, 40, 41, 43, 44, 46, 47, 49, 50, 52, 56–58, 60]. Nineteen studies involved ease of use, about medical services and system technology [17, 20, 23, 27, 29, 30, 32, 36–38, 44–47, 50, 54, 56, 57, 60]. Twenty-five studies involved system quality, which includes telemedicine systems support during telemedicine visits [17, 20, 22, 24, 26–29, 32, 33, 36, 38, 42, 44–47, 50, 54–60]. Nineteen studies involved usefulness, which includes the benefits of telemedicine [17, 20, 22, 24, 26, 32, 34, 36–38, 44, 46, 47, 49, 50, 54–57]. Eight study involved privacy and security, which includes the security of personal privacy when patients use telemedicine [26, 41, 42, 44, 48, 50, 57, 59]. Thirty-nine studies involved overall satisfaction, which is the patient’s general evaluation on telemedicine [17–39, 41–44, 47–52, 54, 56–60].

**Fig. 3:**
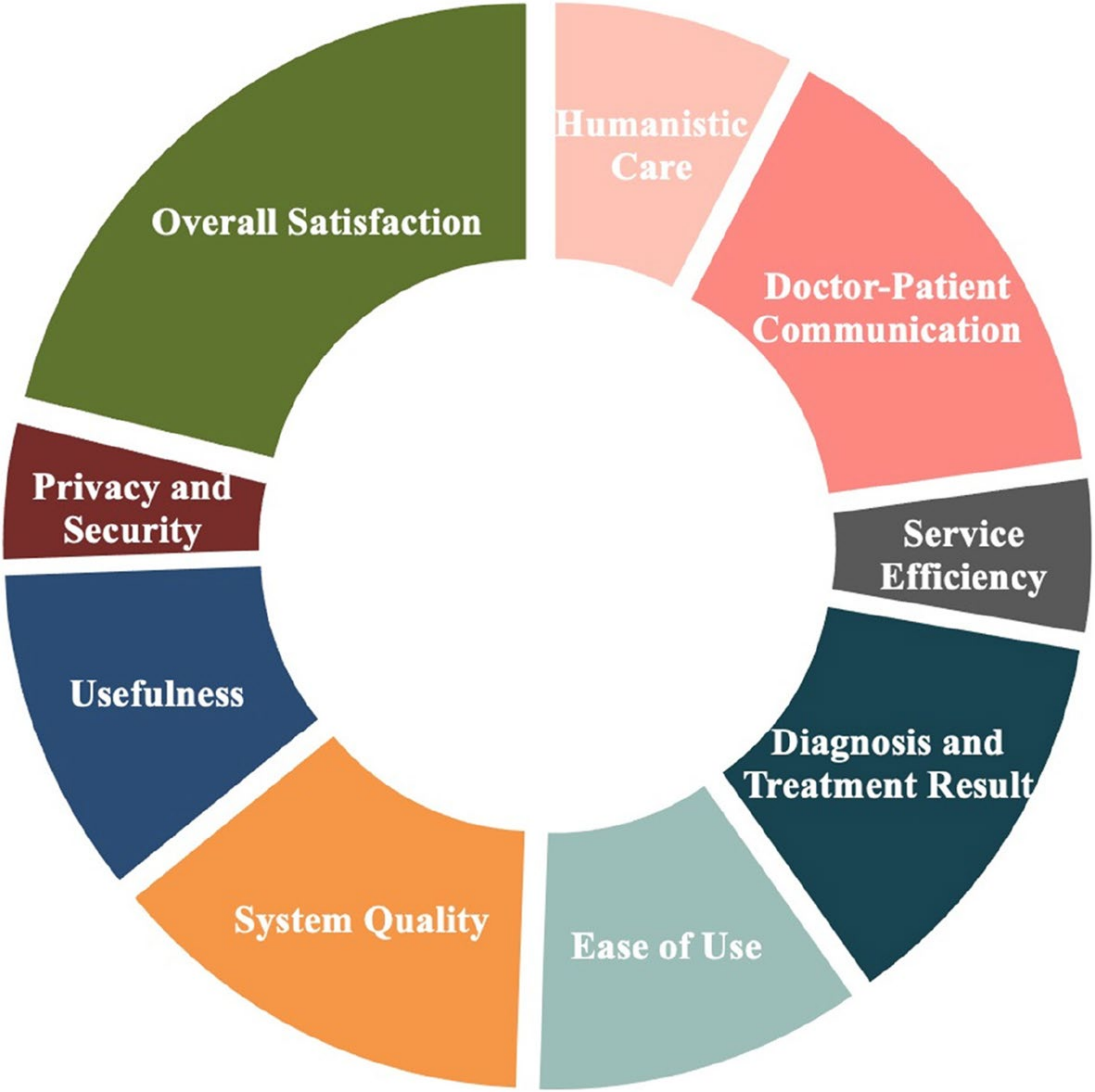
Evaluation scale framework for patient satisfaction with telemedicine

Trough the above work, we developed a systematic patient satisfaction scale for telemedicine, involving 9 dimensions and 37 items. It has 36 objective questions and 1 subjective question. The patient satisfaction conceptual framework and scale for telemedicine are displayed in Fig. 3 and Table 3.

**Table 3:** Patient satisfaction scale for telemedicine

Dimensions and Items	References	Frequency
**Humanistic Care**	[24, 26, 32, 34, 37, 38, 40, 43, 44, 47, 51–53, 56]	**14**
My doctor is courteous.	[44, 51, 53, 56]	4
My doctor is warm and friendly.	[26, 34, 38, 51, 56]	5
My doctor cares about me.	[24, 32, 34, 37, 40, 43, 47, 52, 56]	9
**Doctor-Patient Communication**	[17, 18, 20, 22–24, 26, 29–32, 34, 36, 38, 41–44, 46, 47, 49, 51, 53, 55, 56, 58–60]	**28**
My doctor listens carefully.	[26, 44, 47, 53]	4
My doctor gives me a clear and understandable explain.	[20, 22, 26, 30, 31, 34, 43, 44, 46, 51, 59, 60]	12
My doctor explains diagnosis and treatment in a clear and understandable way.	[26, 34, 38]	3
My medical staff is skillful and knowledgeable.	[18, 32, 38, 47, 51, 53, 56]	7
My doctor asks if I have any questions.	[17, 23, 38, 41, 44]	5
The communication with my doctor is smooth.	[36, 38, 41, 42, 49, 58]	6
There is enough time to communicate with my doctor.	[18, 22, 24, 26, 29, 44, 51, 55, 60]	9
**Service Efficiency**	[17, 29, 41, 42, 47, 53, 58–60]	**9**
My telemedicine visit begins on time.	[29, 42, 47, 53, 58–60]	7
My prescriptions and orders are placed without delay.	[42, 53]	2
I believe I could become productive quickly using telemedicine system.	[17, 41]	2
**Diagnosis and Treatment Result**	[17–19, 23–25, 27, 28, 32, 36, 38, 40, 41, 45–47, 49, 50, 52, 53, 55, 56, 58]	**23**
The telemedicine can solve my every medical problem.	[18, 24, 38, 40, 47, 49, 52, 53, 55]	9
The telemedicine can address my every medical concern.	[19, 28, 50, 53]	4
The telemedicine can satisfy my every medical need.	[17, 23, 25, 27, 28, 32, 36, 41, 45–47, 53, 56, 58]	14
**Ease of Use**	[17, 20, 23, 26, 30, 32–34, 36, 41–43, 45, 47, 53–56, 58]	**19**
The appointment for medical treatment is easy.	[20, 26, 34, 43, 53, 58]	6
The telemedicine system easy to learn.	[17, 23, 41, 56]	4
The telemedicine system easy to use.	[17, 23, 30, 32, 33, 36, 41, 42, 45, 47, 53–56]	14
**System Quality**	[17, 20, 21, 23, 25, 27, 29–33, 36, 37, 41, 42, 45–47, 51, 53–58]	**25**
The quality of system is good.	[17, 21, 37, 41, 42, 53, 55, 56, 58]	9
I can see the doctor clearly.	[17, 20, 23, 27, 29, 31–33, 36, 41, 42, 45–47, 51, 54, 56]	17
I can hear the doctor’s voice clearly.	[17, 20, 23, 27, 29, 31–33, 36, 41, 42, 45–47, 51, 54, 56]	17
I feel comfortable seeing and communicating with the doctor using system.	[17, 20, 23, 25, 27, 30–32, 36, 37, 46, 47, 56, 57]	14
**Usefulness**	[17, 20, 21, 23, 25, 27, 29, 32, 36, 38, 40–43, 45, 47, 53, 55, 56]	**19**
The telemedicine visit saves me travel time.	[17, 21, 23, 27, 32, 36, 40–42, 47, 55, 56]	12
The telemedicine is an acceptable way to receive healthcare services.	[17, 23, 25, 32, 36, 45, 47, 56]	8
The telemedicine visit improves my access to healthcare services.	[17, 20, 23, 25, 29, 32, 40, 41, 45, 47, 55, 56]	12
It is easy to access the telemedicine doctor I need.	[47, 53]	2
I am told what to do when my symptoms get worse.	[38, 43]	2
**Privacy and Security**	[29, 41, 45, 50, 51, 53, 57, 60]	**8**
I am worried about my privacy.	[29, 41, 45, 50, 51, 53, 57, 60]	8
**Overall Satisfaction**	[17–20, 22–43, 45–48, 50–53, 56–60]	**39**
I am satisfied with the health care quality.	[20, 31, 40, 46, 47, 53, 56]	7
I like using this telemedicine system.	[17, 23, 36, 41, 42]	5
Overall, I am satisfied with telemedicine system.	[17, 23, 25]	3
Overall, I am satisfied with telemedicine visit.	[19, 20, 24, 27, 32, 33, 35–38, 42, 45, 46, 48, 50–53, 57–60]	22
My telemedicine visit is as good as in-person visit.	[17, 28, 30, 31, 33, 37, 41, 45, 59]	9
I would use telemedicine services again.	[17, 18, 20, 23–25, 28–33, 35–37, 40, 42, 45, 47, 48, 50–53, 56–59]	28
I would recommend the telemedicine option to other patients.	[22, 26, 27, 30, 33, 34, 42, 43, 51, 59]	10
Expectation of improvement.	[24, 33, 39]	3

## Discussion

Our review of 44 studies that assessed patient satisfaction with telemedicine shows that there is minimal agreement on their evaluation tools. As a result, this study makes a comprehensive questionnaire for future research of patient satisfaction with telemedicine. To our knowledge, this is the first systematic review to develop a scale to assess patient satisfaction with telemedicine by a review and integrate of included studies.

### Summary of included studies

By observing the characteristics of included studies, we found that most of them were from developed countries, including United States, Italy, Spain, Australia, United Kingdom, Canada and France. Although telemedicine has not been popularized worldwide [64], the patient satisfaction survey could provide a reference for developing and underdeveloped countries. In addition, this study contains a variety of disease types, which shows that the future development of telemedicine is almost unlimited by disease types, and the prospect is extremely bright. As a major role in the development of medical services in the future, telemedicine needs to be continuously improved according to patient satisfaction [65].

### Principal findings

In our scale, there was significant variation in the number of reference studies for each dimension (Table 3), 4 dimensions from 20 or more included studies and 3 dimensions from 10 to 20 included studies, while 2 dimension from less than 10 included studies.

For “Humanistic Care” and “Doctor-Patient Communication”, some included studies (*n* = 14) (*n* = 28) supported these 2 dimensions, which could reveal that the strongly importance and closely relevance of these 2 dimensions. The process of establishing interpersonal relationships usually relies on the initial minutes of a conversation [66], including physician’s courtesy, friendliness and care, which determines the patient’s first impression and whether they are willing to trust and communicate with the physician. However, doctor-patient communication is the primary action for the physician and the patient exchange information [67], which involves physician’s listen and explanation. Undoubtedly, doctor-patient communication plays a decisive role in the follow-up close cooperation, diagnosis and treatment, and overall satisfaction.

A small amount of included studies (*n* = 9) discussed the dimension of “Service Efficiency”. Each item of this dimension revolves around decreasing wait times and increasing visit efficiency. It is one of advantages why patient embrace telemedicine [68].

According to included studies (*n* = 23) which relate to the dimension of “diagnosis and treatment result”, whether the medical problem and need were overcame are valued. As the initial expectation of patient who use telemedicine, physical condition improvement closely associates with satisfaction [69]. To be widely adopted, telemedicine must compete favorably with in-person visits in medical outcomes [70].

A number of included studies has the dimension of “Ease of Use” (*n* = 19). It mainly includes appropriate system setting and access of services. Ease of use of the technology is an important factor that can influence or even determine the intention to use telemedicine [71].

“System Quality” (*n* = 25) is the basic factor of telemedicine technology, which may affect telemedicine visits directly and indirectly. On the one hand, audio quality must be of a sufficiently high standard to make effective communication [72]. On the other hand, doctor may get vague message with unclear video. This phenomenon could lead patients’ treatment delay or diagnostic error.

Based on half of (*n* = 19) included studies, the usefulness of telemedicine involves various aspects, including saving travel time [73], increasing access to care and doctor [74]. In the process of offline medical diagnosis and treatment, patients often encounter the problem of registration difficulties, even the experts. However, the online reservation service of telemedicine can better save the travel time of patients and avoid the situation of making a futile trip.

Although the dimension of “privacy and security” are covered by fewer included studies (*n* = 8), it is a major obstacle to the adoption of telemedicine. Most patients are accustomed to consider medical quality, and service efficiency as main factors in choosing medical services, which result in the neglect of privacy issues. However, once there was problem about privacy, it will seriously affect patients’ impressions and even cause distrust telemedicine. Previous questionnaires nearly did not follow with interest this point [75], our research made up the lack of overlooking the privacy and security.

The dimension of “Overall Satisfaction” is included in most included studies (*n* = 39), which is used to measure patients’ integrated perception. TAM’s originators reasoned that the key to increasing use was to first increase acceptance of technology, which could be assessed by asking users about their future intentions to use the technology [76]. Therefore, a subjective question that “Expectation of improvement” was included in this dimension.

Multiple digital and telecommunication technologies have created an unprecedented opportunity for the field of health [77]. As one of the new technology, telemedicine can offer flexibility and convenience to patients [78]. The drivers for satisfaction stem from the benefit of telemedicine [79]. According to the above discussion, during the process of researching the patient satisfaction with telemedicine, we not only pay more attention to the significant dimension, but also can we not ignore the issues that have not yet attracted concern and patient’s expectation of improvement. These will help providers constantly modify or develop systems widely accepted by users. In a word, the widespread adoption of this scale could help transform telemedicine from a convenience-driven technology into a patient-centered healthcare delivery model.

### Limitations

There are 3 limitations in this study. The first limitation is that our research only included three English databases, it may omit valid literatures for our review. The second limitation is that telemedicine is developing rapidly during the COVID-19 pandemic, and its satisfaction measurement factors may be changed in the future. And the last limitation of this study is that this study just research on telemedicine that delivered through video/audio visits.

## Conclusion

This review reported on 44 studies that focused on patient satisfaction with telemedicine for various disease. We developed a scale for evaluating patient satisfaction with telemedicine by applying multidimensional constructs to capture patient satisfaction comprehensively, which involves nine dimensions, such as humanistic care, doctor-patient communication, service efficiency, diagnosis and treatment result, ease of use, system quality, usefulness, privacy and security, overall satisfaction. This scale could be a meaningful tool for future studies to delve into patient satisfaction with telemedicine. Not only will it provide researchers with a framework for quantitatively analyzing patient feedback, but also it will give telemedicine providers insights into areas where they can improve their services. And eventually, providers create a truly “patient-centered” telemedicine service to better meet the needs of patient.

### Supplementary Information

**Additional file 1.** Search strategy.

## Data Availability

All data generated or analyzed during this study are included in this article.
