# Supplementary material for: The development of evaluation scale of the patient satisfaction with telemedicine: a systematic review
Source: BMC Med Inform Decis Mak. 2024 Feb 1;24:31. doi: 10.1186/s12911-024-02436-z (PMC10832124; doi:10.1186/s12911-024-02436-z)
Supplement: Supplementary file 1 — Additional file 1. Search strategy. [file 12911_2024_2436_MOESM1_ESM.pdf]

Additional file 1. Search strategy

Database 1: PubMed

#	Query
1	telemedicine[MeSH Terms]
2	telehealth[Title/Abstract] OR telecommunication[Title/Abstract] OR teleconferenc*[Title/Abstract] OR videoconferenc*[Title/Abstract] OR video consultation[Title/Abstract]
3	1 OR 2
4	satisfaction[Title/Abstract] OR experience[Title/Abstract] OR perception[Title/Abstract] OR preference[Title/Abstract]
5	3 AND 4

Database 2: EMBASE

#	Query
1	(telemedicine OR telehealth OR telecommunication OR teleconferenc* OR videoconferenc* OR video consultation).ti,ab
2	(satisfaction OR experience OR perception OR preference).ti,ab
3	1 AND 2

Database 3: Web of Science

#	Query
1	TS=(telemedicine) OR TS=(telehealth) OR TS=(telecommunication) OR TS=(teleconferenc*) OR TS=(videoconferenc*) OR TS=(video consultation)
2	TS=(satisfaction) OR TS=(experience) OR TS=(perception) OR TS=(preference)
3	1 AND 2
